# Risk Factors for and Clinical Outcomes of Polymicrobial *Acinetobacter baumannii* Bloodstream Infections

**DOI:** 10.1155/2022/5122085

**Published:** 2022-02-27

**Authors:** Zhenhua Qian, Shufang Zhang, Na Li, Weixing Ma, Kai Zhang, Feizhen Song, Cheng Zheng, Li Zhong, Yesong Wang, Jiachang Cai, Hongwei Zhou, Wei Cui, Gensheng Zhang

**Affiliations:** ^1^Department of Critical Care Medicine, Second Affiliated Hospital, Zhejiang University School of Medicine, Hangzhou, Zhejiang 310009, China; ^2^Department of Critical Care Medicine, Shaoxing Central Hospital, Shaoxing, Zhejiang 312000, China; ^3^Department of Cardiology, Second Affiliated Hospital, Zhejiang University School of Medicine, Hangzhou, Zhejiang 310009, China; ^4^Department of Respiratory Medicine, The First Hospital of Jiaxing (The Affiliated Hospital of Jiaxing University), Jiaxing, Zhejiang 314001, China; ^5^Department of Critical Care Medicine, Shengzhou People's Hospital, Shaoxing, Zhejiang 312000, China; ^6^Department of Critical Care Medicine, Taizhou Municipal Hospital, Taizhou, Zhejiang 318000, China; ^7^Department of Critical Care Medicine, Huzhou First People's Hospital, Huzhou, Zhejiang 312000, China; ^8^Clinical Microbiology Laboratory, Second Affiliated Hospital, Zhejiang University School of Medicine, Hangzhou 310009, China

## Abstract

**Background:**

Although the clinical features of *Acinetobacter baumannii* bloodstream infection are well described, the specific clinical characteristics of polymicrobial *Acinetobacter baumannii* bloodstream infection have been rarely reported. The objective of this study was to examine the risk factors for and clinical outcomes of polymicrobial *Acinetobacter baumannii* bloodstream infection.

**Methods:**

A retrospective observational study was performed from January 2013 to December 2018 in a tertiary hospital. All patients with *Acinetobacter baumannii* bloodstream infection were enrolled, and the data were collected from the electronic medical records.

**Results:**

A total of 594 patients were included, 21% (126/594) of whom had polymicrobial infection. The most common copathogen was *Klebsiella pneumoniae* (20.81%), followed by *Pseudomonas aeruginosa* (16.78%) and *Enterococcus faecium* (12.08%). Compared with monomicrobial *Acinetobacter baumannii* bloodstream infection, polymicrobial *Acinetobacter baumannii* bloodstream infection mostly originated from the skin and soft tissue (28.6% vs. 10.5%, *p* < 0.001). Multivariate analysis revealed that burn injury was independently associated with polymicrobial *Acinetobacter baumannii* bloodstream infection (adjusted odds ratio, 3.569; 95% confidence interval, 1.954-6.516). Patients with polymicrobial *Acinetobacter baumannii* bloodstream infection were more likely to have a longer hospital length of stay [40 (21, 68) vs. 27 (16, 45), *p* < 0.001] and more hospitalization days after bloodstream infection than those with monomicrobial *Acinetobacter baumannii* bloodstream infection [22 (8, 50) vs. 13 (4, 28), *p* < 0.001]. However, no significant difference in mortality was observed between the two groups.

**Conclusions:**

Approximately one-fifth of *Acinetobacter baumannii* bloodstream infections were polymicrobial in this cohort. The main sources were skin and soft tissue infections, and burn injury was the only independent risk factor. Although mortality did not differ between the groups, considering the limitations of the study, further studies are required to assess the impact of polymicrobial (vs. monomicrobial) *Acinetobacter baumannii* bloodstream infection on outcomes.

## 1. Background

Bloodstream infection (BSI) is a major cause of hospital-acquired sepsis and causes approximately 157,000 deaths per year in Europe and more than 79,000 deaths per year in North America [1]. As an important gram-negative bacterium, *Acinetobacter baumannii* (AB) accounts for 9%~35% of all BSIs, with an increasing tendency [2, 3]. Due to increases in antibiotic exposure, invasive operations, and carbapenem resistance, AB has become the most common cause of BSI in critically ill patients [4–6]. The overall mortality rate in patients with AB-BSI ranges widely from 29% to 63% [7–9]. Therefore, AB-BSI has become a major challenge in the clinic due to the rapid spread of multidrug resistance among AB and its high associated morbidity and mortality [9–12].

Most BSIs are monomicrobial, but the trend of polymicrobial BSIs is increasing [13, 14]. According to a recent systematic review and meta-analysis, approximately a quarter (range 0% to >60%, depending on the setting and definition) of AB-BSIs are polymicrobial [15]. Although a higher Acute Physiology and Chronic Health Evaluation II (APACHE II) score and a higher frequency of severe sepsis/septic shock are more often observed in patients with polymicrobial BSI than in those with monomicrobial BSI [16, 17], the difference in the mortality rate between the two populations is controversial [15, 18–20]. Limitations of the available literature include the following: (1) polymicrobial AB-BSI is commonly excluded [15], and (2) available data on the impact of polymicrobial (vs. monomicrobial) AB-BSI on clinical outcomes are conflicting [15]. Furthermore, only one previous study has evaluated the impact of polymicrobial AB-BSI on clinical outcomes [21]; thus, comparisons of the characteristics of and risk factors for polymicrobial vs. monomicrobial infections are lacking [15].

The aim of this retrospective study was to determine differences in the proportions and risk factors for and outcomes of polymicrobial and monomicrobial AB-BSIs.

## 2. Materials And Methods

### 2.1. Patients and Study Design

We reviewed the medical records of patients who were admitted to the Second Affiliated Hospital, Zhejiang University School of Medicine, a 3200-bed tertiary healthcare facility in Hangzhou, China, between January 2013 and December 2018. The present study received human research ethics approval (No. 2019-116) from the Ethics Committee of the Second Affiliated Hospital, Zhejiang University School of Medicine. Due to the retrospective nature of the study, the Ethics Committee determined that no patient consent was required.

We collected all AB-positive blood culture results. At least two blood culture samples were collected (at the same time but from different sites) from each patient at the time of the event. Only the first episode was included for patients with more than one episode of AB-BSI. The exclusion criteria were as follows: (a) age < 18 years old; (b) classification of AB as a nonpathogenic bacterium; or (c) incomplete or missing case data. Common skin contaminants (e.g., *Bacillus* spp., *Corynebacterium* spp., *Micrococcus* spp., *streptococci*, *Lactobacillus* spp., and c*oagulase-negative staphylococci*) were considered pathogens only when they were present in two or more consecutive blood cultures from separate blood draws [22]. If skin contaminants were coisolated with AB from a single blood culture, such infections were classified as monomicrobial.

### 2.2. Data Collection

Documented patient demographics, including age, sex, and dates of hospital admission and discharge, were collected. Medical history data included comorbidities at admission, prior hospitalization before BSI, major surgery, the presence of invasive devices (such as central venous catheters, urinary catheters, or drainage catheters), mechanical ventilation, and haemodialysis within 1 week before BSI onset were reviewed. The following parameters within 24 h before BSI onset, reflecting the severity of disease, were collected: the sequential organ failure assessment (SOFA) score, Pitt bacteraemia score, Charlson Comorbidity Index (CCI) score, and Acute Physiology and Chronic Health Evaluation (APACHE) II score. In the first 24 h following the onset of BSI, inflammatory markers such as white blood cell count, procalcitonin level, C-reactive protein level, and liver and kidney function indicators were measured. Microbiological data, such as species involved in polymicrobial AB-BSI, the likely source of the BSI, and sensitivity to antibiotics were gathered. Primary outcomes (all-cause 14-day and 28-day in-hospital mortality) and secondary outcomes (length of total hospital stay and length of hospital stay after BSI onset) were also collected.

### 2.3. Species Identification and Antibiotic Sensitivity Test

Blood culture was performed by using a BacT/ALERT 3D system (Becton-Dickinson, Sparks, MD, USA) in the microbiology laboratory. Species identification was completed using Bruker Daltonics data analysis software. Antibiotic susceptibility was determined with a VITEK 2 system (Card number: AST-GN16; AST-GP67) or the Kirby-Bauer disk diffusion method (Oxford, UK) according to the recommendations of the Clinical and Laboratory Standards Institute (CLSI) [23]. During the study period, there were no changes in microbiological laboratory techniques.

### 2.4. Definitions

AB-BSI was defined as at least one blood culture positive for AB accompanied by two or more of the following symptoms and signs of infection: fever, hypothermia, tachypnoea, tachycardia, leucocytosis, or leukopenia or other corresponding clinical symptoms and signs and the exclusion of specimen contamination [21, 22, 24]. The onset of AB-BSI was defined as the time when the positive blood culture sample was obtained. Polymicrobial AB-BSI was defined as the isolation of AB and one or more other microorganism from the same blood culture, excluding contamination [21, 25]. The primary infection source of BSI was determined according to the definitions of the Center for Disease Control (CDC) [22]. Nosocomial BSI was defined as the first positive blood culture obtained ≥48 h after hospital admission, with no evidence of infection at admission [7, 14]. Prior exposure to antimicrobial agents was defined as antimicrobial treatment for at least 72 h within 30 days prior to the positive blood culture [26]. Appropriate empiric antimicrobial treatment was defined as at least one antibiotic that was active against the pathogenic microorganisms, as confirmed by an in vitro sensitivity test within 48 h of BSI onset [9]. Multidrug resistance was defined as AB resistance to ≥3 classes of antibiotics (quinolones, extended-spectrum cephalosporins, *β*-lactam/*β*-lactamase inhibitor combinations, aminoglycosides, and carbapenems) [27]. Sepsis and septic shock were defined according to the definition of the International Sepsis Definitions Conference [24].

### 2.5. Statistical Analysis

Statistical analysis was performed using SPSS 23.0 software (IBM Corp, Armonk, NY, USA). Continuous variables are presented as means ± standard deviations if normally distributed and as medians and interquartile ranges (IQRs) if nonnormally distributed. Categorical variables were compared by the Pearson *χ*^2^ test or Fisher's exact test. Variables with a *p* value < 0.2 in the univariate logistic regression analysis were entered into the multivariate logistic regression model to determine the independent variables. In addition, clinical scores, such as the APACHE II score, SOFA score, Pitt bacteraemia score, and CCI score, were examined in the multivariate logistic regression model. We excluded patients without intensive care unit (ICU) admission in the analysis of total ICU residence days and ICU residence days after BSI. All tests were 2-tailed, and *p* < 0.05 was considered significant.

## 3. Results

### 3.1. Demographic Characteristics

**Figure 1:**
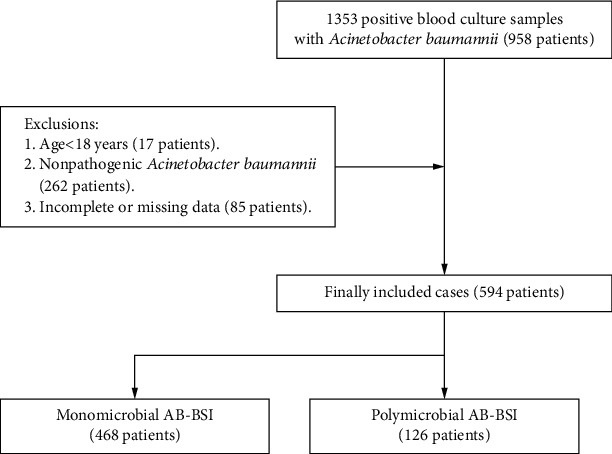
Flowchart of study participant enrollment. Abbreviations: AB-BSI: *Acinetobacter baumannii* bloodstream infection.

From January 2013 to December 2018, a total of 1353 blood culture samples positive for AB were initially identified, and 594 patients with AB-BSI were recruited for analysis. A total of 126 (21%) cases were polymicrobial AB-BSI, and 468 (79%) cases were monomicrobial AB-BSI (Figure 1).

The median patient age was 61 years (IQR, 48-71), and 70.5% were male. The patients with monomicrobial AB-BSI were older than those with polymicrobial AB-BSI (age ≥60 years, 54.5% vs. 44.4%, *p* < 0.05). A total of 96.1% of the patients (571/594) had at least one comorbidity, and a significantly higher rate of burn injury was observed in the polymicrobial AB-BSI group than in the monomicrobial AB-BSI group (23.0% vs. 8.3%, *p* < 0.05). The detailed demographic characteristics of the patients are shown in Table 1.

### 3.2. Biological Indicators

Comparisons of biological indicators between the polymicrobial AB-BSI and monomicrobial AB-BSI groups are shown in Table 2. The glutamic-oxaloacetic transaminase (GOT) level was higher in the polymicrobial AB-BSI group, but there were no significant differences in other liver function indicators or biochemical indicators between the two groups.

### 3.3. Independent Risk Factors for Polymicrobial AB-BSI

Table 3 shows the results of the multivariate logistic regression analysis. Burn injury was the only independent risk factor for polymicrobial AB-BSI (adjusted odds ratio [aOR], 3.569; 95% confidence interval [CI], 1.954-6.516).

### 3.4. Aetiologic Agents of BSIs

**Figure 2:**
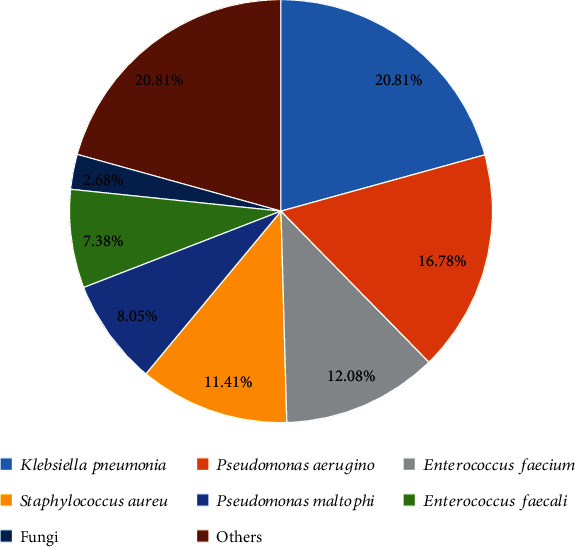
Distributions of coinfecting organisms in polymicrobial *Acinetobacter baumannii* bloodstream infections. Fungi*: Candida albicans*, *Candida near smooth*, and *Candida tropicalis*; others: *Escherichia coli*, *Proteus mirabilis*, *Enterobacter cloacae*, *Serratia marcescens*, etc.; Abbreviations: AB-BSI: *Acinetobacter baumannii* bloodstream infection.

In addition to AB, 149 microorganisms were isolated from 126 patients with polymicrobial AB-BSI, with two microorganisms accounting for 84.1% (106/126) and three microorganisms accounting for 15.87% (20/126). Gram-negative bacteria, gram-positive bacteria, and fungi accounted for 67.1%, 30.2%, and 2.7% of polymicrobial AB-BSI infections, respectively. The most common bacterium involved in polymicrobial AB-BSIs was *Klebsiella pneumoniae* (31/149, 20.8%), followed by *Pseudomonas aeruginosa* (25/149, 16.8%) and *Enterococcus faecium* (18/149, 12.1%). A detailed description of the isolated microorganisms is shown in Figure 2.

The source of AB-BSI was mainly respiratory tract infection (25.9%, 154/594), followed by primary BSI (22.4%, 133/594) and central venous catheter-related infection (15%, 89/451). Compared with those of monomicrobial AB-BSI, the sources of polymicrobial AB-BSI were more frequently skin and soft tissue infections (28.6% vs. 10.5%, *p* < 0.001) and less frequently respiratory tract (15.1% vs. 28.8%, *p* < 0.05) and intracranial infections (2.4% vs. 8.5%, *p* = 0.018) (Table 4).

### 3.5. Antibiotic Resistance and Appropriate Therapy

All BSIs were treated with antibiotics. Antibiotic sensitivity testing showed that there was no difference in sensitivity between the monomicrobial AB-BSI and polymicrobial AB-BSI groups. The resistance rates of AB to ciprofloxacin, ceftazidime, nitrofurantoin, and carbapenems were very high in both groups (more than 90%). In contrary, the ratios of resistance of AB to amikacin, tigecycline, and colistin were relatively low in the two groups (less than 30%). Nine strains of AB resistant to colistin were found in the monomicrobial AB-BSI group, while none was found in the polymicrobial AB-BSI group (Table 4).

A total of 28.5% (169/594) of the patients received appropriate empiric antibiotic therapy. Notably, the rate of appropriate empirical antimicrobial therapy in patients with polymicrobial AB-BSI was substantially higher than that in patients with monomicrobial AB-BSI (38.9% vs. 25.6%, *p* < 0.05).

### 3.6. Outcomes

**Figure 3:**
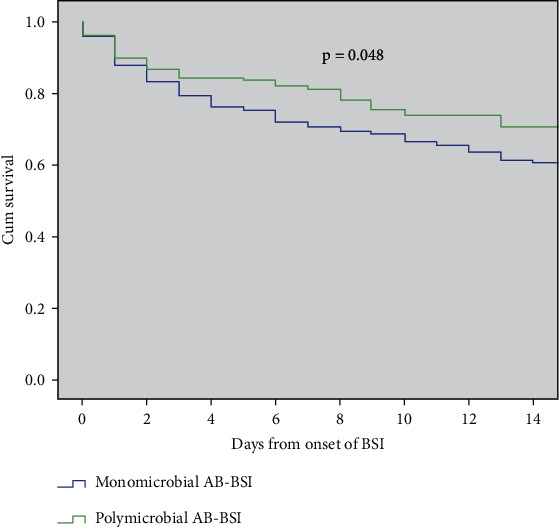
Kaplan-Meier estimates of 14-day survival in patients with polymicrobial AB-BSI and monomicrobial AB-BSI; Abbreviations: AB-BSI: *Acinetobacter baumannii* bloodstream infection.

The prevalence of septic shock was similar (25.4% vs. 28.8%, *p* = 0.445) between the two groups. Patients with monomicrobial AB-BSI had more total ICU residence days [25 (14, 49) vs. 20 (12, 32), *p* < 0.05] and ICU residence days after BSI [11 (3, 33) vs. 8 (3, 18), *p* < 0.05] than those with polymicrobial AB-BSI, while those with polymicrobial AB-BSI had a higher 14-day survival rate (Figure 3) and more total hospitalization days [40 (21, 68) vs. 27 (16, 45), *p* < 0.001] and hospitalization days after BSI onset [22 (8, 50) vs. 13 (4, 28), *p* < 0.001]. However, there were no significant differences in the 14-day and 28-day in-hospital mortality rates between the two groups (Table 5).

## 4. Discussion

The main findings of our study are as follows: (1) a high proportion of AB-BSIs were polymicrobial; (2) gram-negative bacteria (especially *Klebsiella pneumoniae* and *Pseudomonas aeruginosa*) were the most common copathogens, followed by gram-positive bacteria (especially *Staphylococcus aureus* and *Enterococcus* spp.) and fungi; (3) younger age, burn injury, and skin and soft tissue sources of the bacteraemia were associated with polymicrobial (vs. monomicrobial) AB-BSI, and burn injury was the only independent risk factor for polymicrobial AB-BSI; and (4) polymicrobial AB-BSI was associated with longer total and post-BSI hospital stays, but associated mortality rates did not differ significantly.

In the current study, 21% of AB-BSIs were polymicrobial. The range of the proportion of polymicrobial AB-BSI varies widely in the literature (ranging from 0 to >60%), but the estimated pooled proportion in a recent meta-analysis was very close to our findings [15]. Excluding common skin contaminants, gram-negative bacteria were the most common copathogens in polymicrobial AB-BSIs, consistent with the previous findings of the majority of BSIs caused by *Pseudomonas aeruginosa*, *Klebsiella pneumoniae*, and *Staphylococcus aureus* [17, 28, 29]. Similarly, the most common copathogens in mixed-enterococcal BSIs reported in our previous study were gram-negative bacteria (57.1%), followed by gram-positive bacteria (38.3%) and fungi (4.6%) [18]. These results suggest that gram-negative bacteria are the most frequent agents of BSIs and polymicrobial BSIs.

Contrary to other studies [21, 25, 30], there was no difference in the outcome between BSI groups despite a 13.3% higher rate of appropriate empiric antimicrobial treatment in patients with polymicrobial AB-BSI (38.9% vs. 25.6%, *p* < 0.05). The actual rates of appropriate empiric antimicrobial treatment in both groups were low, as both were less than 40%, which might have had a relatively weak impact on the outcomes in patients in both groups. Indeed, rapid pathogen identification and antimicrobial susceptibility testing are crucial for targeted therapy and the initiation of appropriate therapy [2, 31]. However, conventional microbiological processing of a blood culture requires 48-72 h. Molecular rapid diagnostic testing (RDT) has been developed to optimize initial empiric antimicrobial therapy. Nucleic acid amplification tests (NAATs) can identify pathogens approximately 6 h after a blood sample is drawn, and the T2 magnetic resonance (T2MR) assay is a relatively new, fully automated method that can directly detect a range of pathogens in whole blood [32]. However, their clinical performance remains to be validated. RDT plays a prominent role in special pathogen detection, especially in the monitoring of multidrug-resistant gram-negative bacteria [32]. The Amplex eazyplex SuperBug Acineto test can quickly identify carbapenem-resistant AB (CRAB) BSI, and the rapid and simple setup, requiring minimal hands-on time, makes this test suitable for implementation in settings with a high prevalence of CRAB [33]. In addition to conventional blood culture, other rapid diagnostic tools for the detection of AB-BSI should be considered in clinical practice.

In this study, patients with monomicrobial AB-BSI were older and had more tumours than those with polymicrobial AB-BSI. However, the CCI score, APACHE II score, and SOFA score, reflecting the severity of underlying disease, were not different between the groups, similar to the results of a recent study [21]. The only independent risk factor for polymicrobial AB-BSI according to the multivariate analysis was burn injury. Forty-three percent (29/68) of burn patients developed polymicrobial AB-BSI, which was consistent with the results of Tang et al.'s study, which reported that more than 20% of burn patients developed polymicrobial BSI [34]. Burn injury as an independent risk factor for polymicrobial AB-BSI in the current study might partially reflect the significantly higher rates of skin and soft tissue infections as infection sources in the polymicrobial AB-BSI group than in the monomicrobial AB-BSI group (28.6% vs. 10.5%, *p* < 0.05). As described in the previous studies, burn injury can cause the downregulation of cellular and humoral immune responses, extensive disruption of the skin barrier, and gastrointestinal bacterial translocation, resulting in prolonged hospitalization and the need for invasive diagnostic/therapeutic management [35–37]; thus, burn patients have a high risk of BSI. Even though many pathogens colonize the skin, these bacteria are more likely to invade the blood in burn patients, causing polymicrobial BSI, such as polymicrobial AB-BSI in the current study.

The available literature is controversial with regard to the impact of polymicrobial (vs. monomicrobial) AB-BSI on mortality, with most studies showing nonsignificant differences and few studies showing significantly higher or significantly lower mortality rates in polymicrobial AB-BSI patients [15]. According to a recent meta-analysis, polymicrobial AB-BSI was associated with a lower 28-day (but not in-hospital) mortality rate than monomicrobial AB-BSI, which is in agreement with the findings of our study, prompting the hypothesis that other (usually more susceptible) coisolates are the primary pathogens and AB is a secondary pathogen [15]. The larger study sample in the meta-analysis allowed sufficient statistical power to demonstrate differences in mortality between patients with polymicrobial and monomicrobial AB-BSIs [15]. Furthermore, many variables (age, certain comorbidities, appropriate empirical therapy, and source of bacteraemia) were significantly different between polymicrobial and monomicrobial infection patients, limiting the interpretation of comparisons of the two groups.

This study had several limitations: (1) a retrospective design; (2) a small sample size (insufficient power to demonstrate difference in mortality) [15]; (3) a lack of antimicrobial susceptibility testing in all AB isolates; the use of two different antimicrobials susceptibility tests, different specific definitions of AB-BSI, and differences in characteristics between the two groups (polymicrobial vs. monomicrobial groups); (4) heterogeneous polymicrobial infections (the outcomes may differ depending on copathogens and/or sources of bacteraemia) [15]; (5) assessment of all-cause mortality rather than mortality attributable to infection; and (6) lack of consideration of in-hospital mortality and post-BSI length of stay as competing events in the statistical analysis [11]. Taken together, our findings might not be applicable to some other conditions; thus, future multicenter prospective studies are needed.

## 5. Conclusion

Approximately one-fifth of all AB-BSIs were polymicrobial in this cohort. The most common sources of polymicrobial AB-BSI were skin and soft tissue infections, and burn injury was the only independent risk factor for polymicrobial AB-BSI. The post-BSI length of stay was significantly longer in polymicrobial infections patients, but other outcomes (including 14-day, 28-day, and in-hospital mortality) did not differ significantly between the groups. However, considering the limitations of this study, further studies are required to assess the impact of polymicrobial (vs. monomicrobial) AB-BSI on outcomes.

## Figures and Tables

**Table 1:** Demographic and clinical characteristics of monomicrobial AB-BSI and polymicrobial AB-BSI patients.

Characteristics	Total (*n* = 594)	Monomicrobial (*n* = 468)	Polymicrobial (*n* = 126)	*p* value
Age ≥ 60 years	311 (52.4%)	255 (54.5%)	56 (44.4%)	0.046
Male sex	419 (70.5%)	330 (70.5%)	89 (70.6%)	0.979
Comorbidities				
Diabetes mellitus	82 (13.8%)	62 (13.2%)	20 (15.9%)	0.448
Chronic kidney disease	67 (11.3%)	51 (10.9%)	16 (12.7%)	0.571
Chronic liver disease	45 (7.6%)	34 (7.3%)	11 (8.7%)	0.581
COPD^a^ or severe asthma	77 (13%)	59 (12.6%)	18 (14.3%)	0.618
Chronic cardiac insufficiency	130 (21.9%)	98 (20.9%)	32 (25.4%)	0.283
Solid tumour	77 (13%)	68 (14.5%)	9 (7.1%)	0.028
Trauma	146 (24.6%)	119 (25.4%)	27 (21.4%)	0.355
Burn injury	68 (11.4%)	39 (8.3%)	29 (23.0%)	<0.001
Hypertension	190 (32%)	145 (31%)	45 (35.7%)	0.312
Cerebrovascular accident	166 (27.9%)	131 (28%)	35 (27.8%)	0.962
CCI^b^ score, median (IQR)	2 (1,3)	2 (1,3)	2 (0,3)	0.507
APACHE II score, median	18 (13,22)	18 (14,22)	17 (13,22)	0.375
SOFA score, median	6 (4,9)	6 (4,9)	6 (3,9)	0.442
Pitt bacteraemia score, median	4 (3,6)	4 (3,6)	4.5 (3,6)	0.704
Hospitalization ward				
Medical	8 (1.3%)	5 (1.1%)	3 (2.4%)	0.257
Surgical	63 (10.6%)	47 (10%)	16 (12.7%)	0.390
ICU	523 (88%)	416 (88.9%)	107 (84.9%)	0.223
Previous treatment				
Hyperalimentation	227 (38.2%)	176 (37.6%)	51 (40.5%)	0.556
Mechanical ventilation (*n*)	550 (92.6%)	434 (92.7%)	116 (92.1%)	0.798
Surgery	369 (62.1%)	295 (63%)	74 (58.7%)	0.377
Blood transfusion	247 (41.6%)	189 (40.4%)	58 (46.0%)	0.254
Renal replacement therapy	109 (18.4%)	84 (17.9%)	25 (19.8%)	0.626
Carbapenem exposure	237 (39.9%)	182 (38.9%)	55 (43.7%)	0.333
Invasive devices				
Central line	558 (93.9%)	441 (94.2%)	117 (92.9%)	0.566
Indwelling urinary catheter	541 (91.1%)	424 (90.6%)	117 (92.9%)	0.430
Drainage (any site)	253 (42.6%)	197 (42.1%)	56 (44.4%)	0.636
Prior hospital stay, median days (IQR)	11 (7,18)	11 (6,18)	11.5 (7,20)	0.367
Nosocomial infection	559 (94.1%)	438 (93.6%)	121 (96%)	0.301

^a^Chronic obstructive pulmonary disorder. ^b^Charlson Comorbidity Index.

**Table 2:** Comparison of biological indicators between the monomicrobial AB-BSI and polymicrobial AB-BSI groups.

Biochemical indicators	Total (*n* = 594)	Monomicrobial (*n* = 468)	Polymicrobial (*n* = 126)	*p* value
Temperature (°C) (IQR)	38.6 (38.0, 39.1)	38.6 (38.1, 39.1)	38.5 (38.0, 39.03)	0.139
WBC^a^ (×10^9^/L) (IQR)	10.2 (6.9, 14.3)	10.6 (7.03, 14.4)	9.65 (6.65, 13.68)	0.354
Haematocrit (%) (IQR)	25.15 (21.7, 29.93)	25.3 (22.0, 30.48)	24.2 (21.0, 28.33)	0.031
Platelets (×10^9^/L) (IQR)	144 (77.75, 228.25)	147.5 (79, 234.75)	128.5 (69.75, 217.5)	0.340
ANC^b^ (IQR)	8.71 (5.76, 12.77)	9.02 (5.87, 12.93)	8.35 (5.27, 12.10)	0.276
Albumin (g/L) (mean ± SD)	30.65 ± 5.82	30.85 ± 5.82	30.4 ± 5.79	0.201
GPT^c^ (U/L) (IQR)	37 (23, 68)	37 (23, 70)	39 (27.5, 63.25)	0.330
GOT^d^ (U/L) (IQR)	41 (26, 67)	39 (26, 66)	48.5 (28, 75.5)	0.021
TBil^e^ (umol/L) (IQR)	19.6 (12.18, 34.23)	19.6 (12.13, 35.78)	19.65 (12.18, 31.95)	0.787
SCr^f^ (umol/L) (IQR)	62 (46, 99)	63 (46, 100.75)	57 (44.75, 95.25)	0.673
Lactic acid (mol/L) (IQR)	2.1 (1.3, 3.1)	2.1 (1.3, 3.2)	2 (1.28,2.83)	0.461
CRP^g^ (mg/L) (IQR)	119.85 (67.05, 195.5)	120.25 (67.90, 199.75)	119.35 (63.98, 180.0)	0.431
PCT^h^ (ng/mL) (IQR)	1.74 (0.45, 6.53)	1.72 (0.44, 6.71)	1.91 (0.50, 6.31)	0.979

^a^White blood count. ^b^Absolute neutrophil count. ^c^Glutamic-pyruvic transaminase. ^d^Glutamic-oxaloacetic transaminase. ^e^Total bilirubin. ^f^Serum creatinine. ^g^C-reactive protein. ^h^Procalcitonin.

**Table 3:** Logistic regression analysis of factors associated with polymicrobial AB-BSI.

Characteristics	Univariable analysis		Multivariable analysis	
	Unadjusted OR (95% CI)	*p* value	Adjusted OR (95% CI)	*p* value
Age ≥ 60 years	0.668 (0.450, 0.993)	0.046	0.855 (0.546, 1.338)	0.493
Male sex	0.994 (0.646, 1.531)	0.979		
Diabetes mellitus	1.236 (0.715, 2.136)	0.449		
Chronic kidney disease	1.189 (0.653, 2.166)	0.571		
Chronic liver disease	1.211 (0.600, 2.484)	0.582		
COPD^a^ or severe asthma	0.866 (0.490, 1.529)	0.619		
Chronic cardiac insufficiency	0.779 (0.492, 1.231)	0.284		
Solid tumour	1.936 (1.027, 3.651)	0.028	2.120 (0.973, 4.620)	0.059
Trauma	1.250 (0.779, 2.008)	0.355		
Burn injury	2.313 (1.664, 3.214)	<0.001	3.569 (1.954, 6.516)	<0.001
Hypertension	0.808 (0.534, 1.222)	0.313		
Cerebrovascular accident	1.011 (0.652, 1.568)	0.962		
CCI^b^	0.965 (0.869, 1.071)	0.500	1.119 (0.984, 1.274)	0.087
APACHE II score	0.986 (0.956, 1.016)	0.352	0.980 (0.934, 1.029)	0.423
SOFA score	0.991 (0.942, 1.042)	0.721	0.986 (0.925, 1.052)	0.671
Pitt bacteraemia score	1.017 (0.936, 1.104)	0.696	1.083 (0.969, 1.212)	0.162
Medical	0.443 (0.104, 1.878)	0.269		
Surgical	0.768 (0.419, 1.405)	0.391		
ICU	1.421 (0.806, 2.504)	0.225		
Hyperalimentation	0.886 (0.593, 1.325)	0.556		
Mechanical ventilation (*n*)	1.100 (0.528, 2.293)	0.798		
Surgery	1.198 (0.802, 1.790)	0.377		
Blood transfusion	0.794 (0.535, 1.180)	0.254		
Renal replacement therapy	0.884 (0.537, 1.453)	0.626		
Carbapenem exposure	0.821 (0.552, 1.223)	0.333		
Central line	1.256 (0.575, 2.745)	0.567		
Indwelling urinary catheter	0.741 (0.352, 1.562)	0.431		
Drainage (any site)	0.909 (0.611, 1.351)	0.636		
Prior hospital stay	1.005 (0.996, 1.015)	0.293		
Nosocomial infection	0.603 (0.229, 1.588)	0.306		

^a^Chronic obstructive pulmonary disorder. ^b^Charlson Comorbidity Index.

**Table 4:** Comparisons of the microbiological characteristics of monomicrobial AB-BSIs and polymicrobial AB-BSIs.

Antibiotic resistance	Total (*n* = 594)	Monomicrobial (*n* = 468)	Polymicrobial (*n* = 126)	*p* value
Source of BSI				
Respiratory tract	154 (25.9%)	135 (28.8%)	19 (15.1%)	0.002
Central venous catheter	89 (15%)	64 (13.7%)	25 (19.8%)	0.085
Skin and soft tissue	85 (14.3%)	49 (10.5%)	36 (28.6%)	<0**.001**
Intracranial	43 (7.2%)	40 (8.5%)	3 (2.4%)	0.018
Primary	133 (22.4%)	107 (22.9%)	26 (20.6%)	0.594
Others^a^	90 (15.2%)	73 (15.6%)	17 (13.5%)	0.552
Antibiotic resistance of AB^b^				
Amikacin (330 vs. 81)^c^	145 (24.4%)	115 (24.6%)	30 (23.8%)	0.380
Ciprofloxacin (467 vs. 126)^c^	545 (91.8%)	431 (92.1%)	114 (90.5%)	0.701
Ceftazidime (463 vs. 125)^c^	556 (93.6%)	440 (94%)	116 (92.1%)	0.598
Tobramycin (460 vs. 121)^c^	409 (68.9%)	316 (67.5%)	93 (73.8%)	0.068
Levofloxacin (467 vs. 126)^c^	519 (87.4%)	412 (88%)	107 (84.9%)	0.532
Nitrofurantoin (431 vs. 117)^c^	541 (91.1%)	425 (90.8%)	116 (92.1%)	0.864
Cefoperazone/sulbactam (460 vs. 125)^c^	520 (87.5%)	409 (87.4%)	111 (88.1%)	0.756
Gentamicin (455 vs. 121)^c^	461 (77.6%)	358 (76.5%)	103 (81.7%)	0.229
Piperacillin/tazobactam (241 vs. 61)^c^	273 (46%)	217 (46.4%)	56 (44.4%)	0.761
Carbapenems (467 vs. 126)^c^	550 (92.6%)	435 (92.9%)	115 (91.3%)	0.673
Tigecycline (391 vs. 106)^c^	165 (27.8%)	135 (28.8%)	30 (23.8%)	0.475
Colistin (253 vs. 57)^c^	9 (1.5%)	9 (1.9%)	0 (0%)	0.083
Treatment after the onset of BSIs				
Appropriate empiric antibiotic treatment	169 (28.5%)	120 (25.6%)	49 (38.9%)	0.003

^a^Biliary tract, heart surgery, urinary tract, and intraabdominal. ^b^AB: *Acinetobacter baumannii*; not all agents listed tested in all isolates. ^c^The numbers in parentheses represent the total numbers of AB isolates that were subjected to susceptibility testing.

**Table 5:** Comparisons of outcomes between monomicrobial AB-BSIs and polymicrobial AB-BSIs.

Prognostic indicators	Total	Monomicrobial	Polymicrobial	*p* value
Total hospitalization days (M) (IQR)	29 (18, 50)	27 (16, 45)	40 (21, 68)	<0.001
Hospitalization days (M) after BSI (IQR)	14 (5, 32)	13 (4, 28)	22 (8, 50)	<0.001
Total ICU residence days (M) (IQR)	20 (12, 35)	25 (14, 49)	20 (12, 32)	0.003
ICU residence days after BSI (M) (IQR)	9 (3, 20)	11 (3, 33)	8 (3, 18)	0.003
Sepsis (*n*, %)	573 (96.5%)	454 (97%)	119 (94.4%)	0.167
Septic shock (*n*, %)	167 (28.1%)	135 (28.8%)	32 (25.4%)	0.445
14-day mortality (*n*, %)	211 (35.5%)	175 (37.4%)	36 (28.6%)	0.066
28-day mortality (*n*, %)	252 (42.4%)	206 (44%)	46 (36.5%)	0.130
In-hospital mortality (*n*, %)	283 (47.6%)	228 (48.7%)	55 (43.7%)	0.312

## Data Availability

Data can be obtained from the corresponding author.
